# Boosting Intelligent Data Analysis in Smart Sensors by Integrating Knowledge and Machine Learning

**DOI:** 10.3390/s21186168

**Published:** 2021-09-14

**Authors:** Piotr Łuczak, Przemysław Kucharski, Tomasz Jaworski, Izabela Perenc, Krzysztof Ślot, Jacek Kucharski

**Affiliations:** Institute of Applied Computer Science, Lodz University of Technology, Stefanowskiego 18/22, 90-537 Łódź, Poland; pluczak@iis.p.lodz.pl (P.Ł.); pkuchars@iis.p.lodz.pl (P.K.); tjaworski@iis.p.lodz.pl (T.J.); iperenc@iis.p.lodz.pl (I.P.); kslot@p.lodz.pl (K.Ś.)

**Keywords:** AI-enabled sensors, hybrid systems, feedforward neural networks, knowledge embedding

## Abstract

The presented paper proposes a hybrid neural architecture that enables intelligent data analysis efficacy to be boosted in smart sensor devices, which are typically resource-constrained and application-specific. The postulated concept integrates prior knowledge with learning from examples, thus allowing sensor devices to be used for the successful execution of machine learning even when the volume of training data is highly limited, using compact underlying hardware. The proposed architecture comprises two interacting functional modules arranged in a homogeneous, multiple-layer architecture. The first module, referred to as the knowledge sub-network, implements knowledge in the Conjunctive Normal Form through a three-layer structure composed of novel types of learnable units, called L-neurons. In contrast, the second module is a fully-connected conventional three-layer, feed-forward neural network, and it is referred to as a conventional neural sub-network. We show that the proposed hybrid structure successfully combines knowledge and learning, providing high recognition performance even for very limited training datasets, while also benefiting from an abundance of data, as it occurs for purely neural structures. In addition, since the proposed L-neurons can learn (through classical backpropagation), we show that the architecture is also capable of repairing its knowledge.

## 1. Introduction

In recent years, remarkable improvement has been shown in both the capabilities and efficiency of intelligent systems [1], yet the state-of-the-art models continue to grow in size. Not only are intelligent systems now capable of achieving state-of-the-art performance on multiple complex games, as shown by AlphaZero [2], but they are also capable of solving extremely complex real-world problems such as protein folding. The most recent release of AlphaFold [3] proved to be capable of solving the 14th Critical Assessment of protein Structure Prediction (CASP) challenge [4], thus providing an invaluable tool for modern bioinformatics research. These performance improvements are achieved at the expense of increases in model size, such as in the case of the GPT (Generative Pre-trained Transformer) family of models that went from 1.5 billion parameters in 2019 [5] to 175 billion parameters in 2020 [6]. These large models, while still feasible to train thanks to algorithmic and technological advances, require ever-increasing amounts of input examples, which may be unavailable, especially when application-specific tasks, typical for smart sensors, are considered. In addition, implementing large neural networks on resource-limited devices is infeasible, so if machine learning is to be considered as a problem-solving strategy for smart sensors, one needs to look for network complexity reduction concepts that preserve a sufficient capacity for handling real-world problems.

Since large neural models learn everything from scratch, a significant part of training time is spent learning relations that are inherently obvious to a human expert. A possible remedy to this problem is introducing expert knowledge into training, for example, through appropriate initialization, placing additional constraints, or arranging a network structure that provides general scaffolding for the model. This scaffolding could also help to alleviate the ever-growing concern with the safety of AI [7], potentially providing a novel approach to understanding the model [8] and eventually leading to the development of genuinely Explainable Artificial Intelligence (XAI) [9]. The development of such algorithms would enable machine learning agents to be deployed in solving critical tasks such as decision making in medical applications [10] or the dynamic reconfiguration of critical telecommunication infrastructure [11].

In the presented paper, we propose a novel hybrid neural architecture that can be considered for implementation in resource-limited devices and which combines learning from examples with expert knowledge using two interacting functional modules arranged in a homogeneous, multiple-layer architecture. A core element of the proposed concept is a novel model of a neuron, which we refer to as *Logic-neuron* (L-neuron), which embeds propositional logic expressions and integrates seamlessly into a conventional, feed-forward multiple-layer neural architecture, both during network training (using the classical backpropagation approach) and during normal operation. We show that the insertion of the proposed knowledge-embedding module—layered ensembles of L-neurons—into a conventional multiple-layer structure significantly improves the performance of a network trained on limited datasets (i.e., that embedded knowledge compensates a lack of sufficient training data). As the proposed hybrid network compensates learning with knowledge, its conventional neural part only needs to learn partial information on the considered problem, so it can become simpler and thus, easier to implement. Moreover, we also show that the proposed module is capable of fixing impaired knowledge during training by adjusting or ignoring erroneous predicates of logical expressions or by refining logical term aggregation rules.

We begin the paper with a short review of relevant work. Then. we provide detailed information on the proposed concept: L-neuron definition, the description of network architecture and training loss formulation. Finally, we present concept evaluation using a sample problem of one-dimensional data analysis, together with a discussion of results and concluding remarks.

## 2. Related Work

Numerous attempts to seamlessly combine knowledge with neural networks have been made since their inception. Some of them took advantage of the universal approximation property of neural networks [12,13,14] and focused on providing a more complete description of the approximated function than what could be provided by the samples alone. A very notable family of such approaches has been developed by Gori et al. [15] by providing knowledge representation in the form of constraints injected as additional components of a loss function. Roychowdhury et al. [16] present a way of constructing a graph of logical expressions through which the error can be propagated in both directions in order to create a constraint function that closely resembles a knowledge base. As flexible as they are in terms of knowledge formulation, these approaches lack verifiability of both knowledge inclusion and conservation after the network’s training settles down.

An intuitive approach for inserting knowledge into a network is to configure its structure to resemble the knowledge base. Such a configuration can either be prepared manually [17] or generated in the training process [18]. Numerous attempts at preloading networks with knowledge have been proposed by Towell et al. [17,19], where nodes of a network form a graphical model that represents knowledge, while logical operations carried out by each node are set by the appropriate choice of weights and biases. Such a network can be subsequently trained in a standard way, and it proved to be capable of knowledge refinement [20]. Once trained, the network’s weights and biases can, at least to some degree, be translated back into rules. Compared to previous propositions, it benefits from the control over initial knowledge incorporation but falls short on built-in mechanisms of knowledge preservation in the trained model.

A Logical Neural Network (LNN) [21] maintains a one-to-one correspondence between neurons and elements of logical formulas, but it does not enable logical and data-driven knowledge to be combined. In the case of Relational Neural Machines (RNMs) [22], knowledge is provided through optimization constraints, thus being a part of the parametric regularization and not of the learner structure. In Deep Reasoning Networks [23], prior knowledge is inserted into the network’s latent space in the form of constraints. The method proposed by Herrmann and Thier [24] includes a set of neurons performing logical disjunction and conjunction operations. However, to satisfy the constraints of such operations, a special backpropagation method is introduced, which, apart from clipping the weights to the desired range, also applies the winner-takes-most (WTM) weight-update strategy.

An alternative approach to building knowledge-enabled neural models has been proposed by Koh et al. [25]. An interpretable latent representation (a ‘bottleneck’) trained to comprise high-level bird visual attributes (e.g., wing color, beak color, etc.) was used for decision making. Using their expert knowledge, a user was able to validate final predictions against a particular bottleneck’s neurons activation patterns, as well as correct them to better describe the contents of the input image. This approach, while extremely promising in terms of result interpretability, requires human involvement, so the proposed concept is semi-automatic only.

Methods of building knowledge structure through training on available samples have seen development as recently as 2019 [18]. The WANN approach is based on NEAT [26] and involves the gradual, evolutionary growth of a network in order to obtain a structure that fits the problem. Whereas the NEAT algorithm optimizes both network weights and structure simultaneously, WANN simplifies the problem by only optimizing a structure, using a single weight for all nodes. This approach can be seen as a gradual construction of a knowledge base. The fundamental incompatibility with classical, layered neural networks makes WANNs difficult to integrate with methods developed for ANNs. The SATNet approach [27] focuses on learning a logical structure based on samples, with no expert knowledge preloaded to the model.

An entirely separate category of solutions is represented by the ANFIS model [28], which provides a way of refining fuzzy knowledge based on the available data. Unlike other approaches described in this section, this technique is not based on neural networks but instead explicitly operates on fuzzy membership functions. An obvious shortcoming of the ANFIS approach is the requirement to represent the whole solution explicitly. Despite its inherent limitations, the ANFIS model has found many uses in both control and classification tasks.

## 3. Materials and Methods

In intelligent systems with logic-based knowledge representation, the formulas are usually simplified to clauses κ consisting of literals, i.e., statements and negated statements. It has been proven that every logical formula can be transformed into a set of clauses connected by conjunctions, which yields its *Conjunctive Normal Form (CNF)*. Hence, each knowledge base, *KB*, can be presented in the form:

(1) $$\mathrm{KB}=\left\{\kappa_{1}\wedge \kappa_{2}\wedge \cdots \wedge \kappa_{n}\right\}\equiv \left\{\kappa_{1},\kappa_{2},\cdots ,\kappa_{n}\right\}.$$

This allows for any knowledge base to be implemented by using only *negation*, *conjunction* and *disjunction* operators. It is also worth noting that the clause consisting of sets of $p_{i}$ and $q_{j}$ logical statements:

(2) $$p_{1}\vee p_{2}\vee \cdots \vee p_{l}\vee \neg q_{1}\vee \neg q_{2}\vee \cdots \neg q_{k}$$

can be rewritten as an implication expression:

(3) $$q_{1}\wedge q_{2}\wedge \cdots \wedge q_{k}\to p_{1}\vee p_{2}\vee \cdots \vee p_{l}$$

which directly realizes the *if...then* conditional statement, and which is typically used to formulate the expert knowledge expressing causal relations between different entities of the problem.

### 3.1. L-Neuron

**Figure 1:**
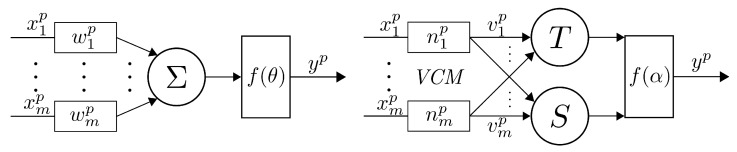
Architectures of the proposed L-neuron (**right**) and Pitts–McCulloch models (**left**) feature a similar dataflow pipeline: input transformation (weighting vs. VCM), aggregation (scalar vs. vectorized, through *T*- and *S*-norms) and scalar output generation (nonlinear function vs. linear mixing).

The principal component of our approach is a novel model of a knowledge processing unit, referred to as an L-neuron (Figure 1), with architecture resembling the Pitts–McCulloch neuron model [29].

To enable the flexible implementation of logic terms, L-neurons need to enable recruiting input variables (crisp or fuzzy) either in their original or complemented forms. They should also be able to discard these variables if they appear irrelevant. This functionality needs to be trainable, so one needs to elaborate an appropriate, continuously differentiable parametric function that appropriately preprocesses each L-neuron’s input. The proposed *variable conditioning module* (VCM, see Figure 1) transforms an input variable, $x_{i}$, into a logic-term literal, $v_{i}$, using a function defined as:

(4) $$v_{i}\left(n_{i},x_{i},z\right)=\phi \left(n\right)x_{i}+\psi \left(n\right)+\xi \left(n\right)z$$

where $n\in [n_{min},n_{max}]$ is a control parameter, *z* is a binary value representing a neutral symbol for either of the two logic operators: for conjunction or its fuzzy extension—*T*-norm, z=1 (as T(x,z=1)=x, where T(.) denotes binary *T*-norm operation on variables *x* and *z*), whereas for disjunction, or *T*-conorm (*S*-norm) z=0 (as S(x,z=0)=x). Inclusion of the parameter *z* enables one to ignore irrelevant input variables. The symbols ϕ(·),ψ(·),ξ(·) denote some parametric functions that provide the continuity and differentiability of (4), as well as enforce the fulfillment of transformation bounds. These bounds are defined as follows: for the two extreme control parameter values, the function is to implement either negation ($v_{i}\left(n_{\max},x_{i},z\right)\;=\;1-x_{i}$) or passthrough operation ($v_{i}\left(n_{\min},x_{i},z\right)\;=\;x_{i}$), whereas for the central value of the control parameter ($n_{mean}\;=\;0.5\left(n_{\min}+n_{\max}\right)$), the transformation should produce a neutral element ($v_{i}\left(n_{mean},x_{i},z\right)\;=\;z$).

Variables involved in prior knowledge expressions are transformed by VCM units with control parameters initialized to either of the extreme values ($n=n_{min}$ or $n=n_{max}$). Since our knowledge may be incomplete, the remaining variables can be linked to each L-neuron in a neutral manner ($n=n_{mean}$). Changing VCM control parameters during training enables knowledge extension (by adopting inputs that were initially not included in logical terms) or knowledge repair (by altering variable forms or removing variables from terms).

The proposed control mechanism does not guarantee logic axioms satisfaction neither during learning nor after training is completed, as a variable can be biased towards “neutrality” (if $n\in (n_{min},n_{mean})$ or if $n\in (n_{mean},n_{max})$). However, this divergence is permanently restricted by logically valid concepts, protecting knowledge from being wholly erased during training. Furthermore, "relaxed" (biased towards neutrality) forms of negation or passthrough can be seen as means for reflecting uncertain knowledge.

**Figure 2:**
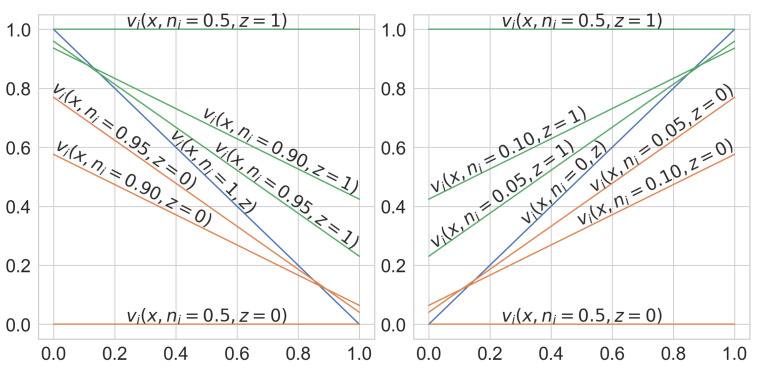
Sample transformations of input variables for control parameters (**left**) $n\geq n_{mean}$ (“negation” mode); (**right**) $n\leq n_{mean}$ (“passthrough” mode).

**Figure 3:**
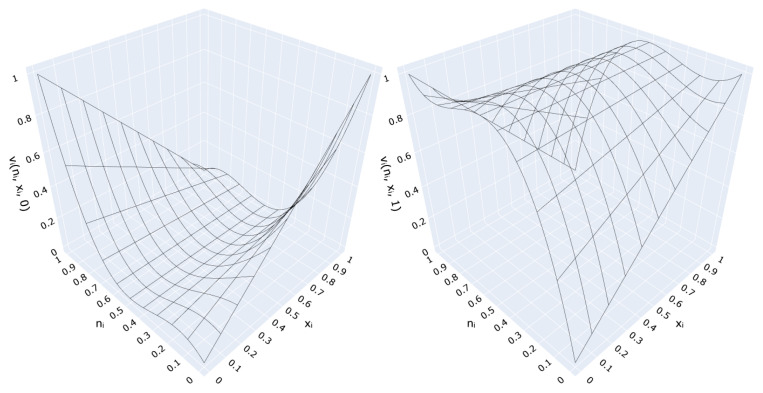
VCM transformation (**left**) z=0 (*T*-conorm neutral value); (**right**) z=1 (*T*-norm neutral value).

The simplest possible candidates for continuous and differentiable VCM transformations (4), which enable gradient-based training, are second-order polynomials. However, in order to minimize divergence from logic principles, we also want to keep the result of the transformation (4) bounded within a legitimate range of logic values (i.e., between 0 and 1) at all times. Therefore, throughout the remaining research, we adopted a slightly more complex VCM form, involving the third-order polynomial:

(5) $$v_{i}=-{\left(2n_{i}-1\right)}^{3}x_{i}+{\left(1-2n_{i}\right)}^{2}n_{i}+\left(-4n_{i}^{2}+4n_{i}\right)z$$

where the parameter *n* range is from $[n_{min}\;=\;0$ to $n_{max}\;=\;1]$. Linear transformations of input variables provided by (5) for selected values of the control parameter and three different modes (complement, passthrough and neutral) are shown in Figure 2, while the complete transformation is depicted in Figure 3.

The gradient of (5) with respect to $n_{i}$ has a form:

(6) $$\frac{\partial v_{i}\left(n_{i},x_{i},z\right)}{\partial n_{i}}=-6x_{i}{\left(2n_{i}-1\right)}^{2}-4n_{i}\left(1-2n_{i}\right)+{\left(1-2n_{i}\right)}^{2}+z\left(-8n_{i}+4\right).$$

Since *z* is constant (either z=0 or z=1), the two possible forms of (6) are:

(7) $$\frac{\partial v_{i}\left(n_{i},x_{i},1\right)}{\partial n_{i}}=-6x_{i}{\left(2n_{i}-1\right)}^{2}-4n_{i}\left(1-2n_{i}\right)+{\left(1-2n_{i}\right)}^{2}+\left(-8n_{i}+4\right)$$

and

(8) $$\frac{\partial v_{i}\left(n_{i},x_{i},0\right)}{\partial n_{i}}=-6x_{i}{\left(2n_{i}-1\right)}^{2}-4n_{i}\left(1-2n_{i}\right)+{\left(1-2n_{i}\right)}^{2}.$$

**Figure 4:**
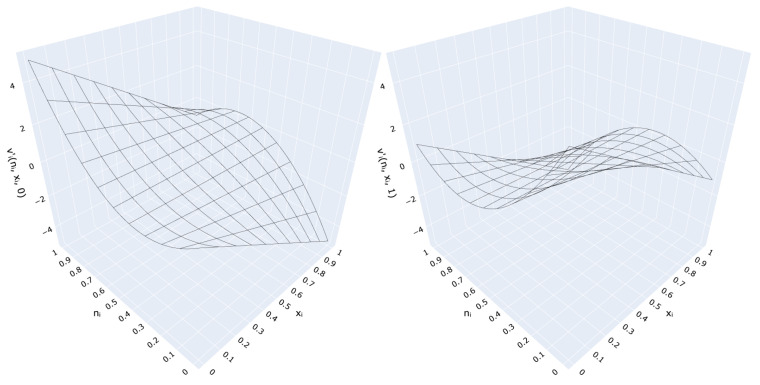
Gradient of the considered VCM transformation (**left**) for z=0 and (**right**) for z=1.

Gradient distributions for both *T*-conorm neutral value and *T*-norm neutral value are depicted in Figure 4. Observe that local minima at $n_{min}=0$ and $n_{max}=1$ drive gradient-based optimization towards either “passthrough” or “negation” configurations of the VCM. As a consequence, it can be asserted that the application of standard neural network optimization algorithms, such as Stochastic Gradient Descent or Adam, which are known to be convergent [30], results in training convergence for the proposed model.

Preprocessed input values are subsequently evaluated by means of fuzzy *T*-norm and *T*-conorm. Since *T*-norms and *T*-conorms are binary operators, the following recursive extensions can be applied to enable the evaluation of multiple-element vectors $v_{m\times 1}$:

(9) $$\begin{array}{cc} T(v) & :T^{\prime }(v_{1},T^{\prime }(v_{2},\cdots T^{\prime }\left(v_{m-2},T^{\prime }\left(v_{m-1},v_{m}\right)\cdots \right)\\ S(v) & :S^{\prime }(v_{1},S^{\prime }(v_{2},\cdots S^{\prime }\left(v_{m-2},S^{\prime }\left(v_{m-1},v_{m}\right)\cdots \right). \end{array}$$

Whilst binary $T^{\prime }$ and $S^{\prime }$ functions could, in theory, be any pair of differentiable *T*-norm and *T*-conorm, it has been observed that the *algebraic* [31] norms (10) provide the best results.

(10) $$\begin{array}{cccc} T^{\prime }\left(v_{1},v_{2}\right) & =v_{1}\cdot v_{2} & \; & \\ S^{\prime }\left(v_{1},v_{2}\right) & =v_{1}+v_{2}-v_{1}\cdot v_{2}. & \; \end{array}$$

The results of *T*-norm and *T*-conorm evaluation, performed by each L-neuron, are subsequently linearly combined in the aggregation block:

(11) f(v,α)=αT(v)+(1−α)S(v)

where α∈[0,1] is the conjunction/disjunction bias of the neuron and $v\in {\left[0,1\right]}^{m}$ is the output of the VCM. The aggregation block f(.) provides a smooth transition between the AND and OR operators and resembles a uni-norm [32], while being easier to interpret and configure. At the beginning of training, all L-neurons are initialized with crisp values (either 0 or 1) of the α parameter, reflecting the CNF formulation of the initial knowledge. However, the optimization algorithm is free to change norm-mixing proportions, and after training is completed, the initial configuration can even be completely inverted. A clear benefit of this approach is the ability to embed uncertain knowledge in which the expert may be unsure of the exact nature of the described relations.

Our formulation of the L-neuron attempts to provide seamless integration with classical neurons and gradient-based learning methods. However, satisfying this objective limits the range of available *T*-norms and *T*-conorms, as it disallows the use of Zadeh, Bounded, Fodor, or Drastic [31] norms due to non-differentiability of the underlying minimum and maximum operator definitions.

Since all L-neuron operations are defined in terms of addition and multiplication (either element-wise or matrix), its formulation is differentiable and thus compatible with all gradient-based learning methods. This property, in turn, renders our contribution directly compatible with existing neural frameworks, such as PyTorch or TensorFlow, for both training and inference.

### 3.2. Proposed Network Architecture

**Figure 5:**
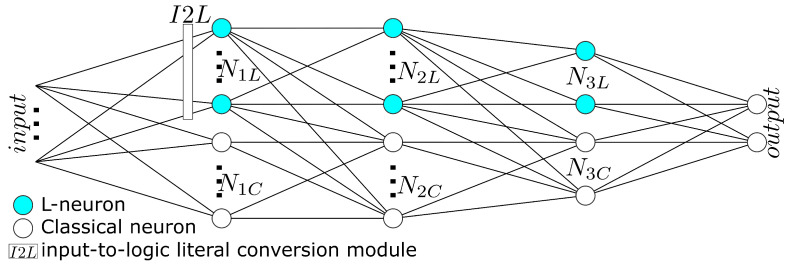
Architecture of the proposed hybrid network.

The proposed hybrid architecture comprises two sets of processing units: L-neurons and classical neurons, arranged in a multiple-layer, feed-forward-only structure, presented in Figure 5. Interconnections between the two types of units are non-symmetric: classical neurons are fed with outputs produced by preceding layer neurons of both types, yet L-neurons are fed only by preceding knowledge units. This asymmetry isolates knowledge from concepts learned by conventional neurons, which are not suitable for ‘logical’ interpretation, enabling rule explainability at any phase of training. The outcome produced by the two processing modules, the L-neuron-based knowledge sub-network and the conventional neural sub-network, is merged at the last layer comprising classical neurons with dense input connection. These neurons linearly combine information produced by the two processing pipelines and generate the network’s output.

All neurons of the proposed hybrid network are trained using the backpropagation algorithm. To emphasize the significance of inserted knowledge, weights of output-layer neurons are manually initialized to favor the importance of decisions produced by the knowledge module (initial couplings with knowledge module outputs are significantly stronger).

As logical expressions operate on concepts rather than on raw data, a ‘concept extraction’ unit needs to be used to transform input to the knowledge sub-network. This can be carried out by input fuzzification (similar to the ANFIS paradigm) or by implementing any other relevant input data aggregation rules that produce logic literals. Many sensors deployed in edge computing devices are well suited for direct data fuzzification, as they are fed with scalar measurements. This approach can also be applied in more complex solutions, such as the intelligent reconfiguration of HMIMOS [11] or AI-assisted methods described by Cao et al. [33]. In situations where the classical fuzzy-logic approach for input conversion cannot be applied, an alternative approach for concept extraction proposed by Koh et al. [25] can be used in order to generate logically tractable values. Since classical neurons are also fed with outputs from L-neurons that are restricted within the [0…1] interval, all classical neurons use sigmoid activations to ensure the compatibility of both sources.

### 3.3. Loss

The proposed loss, *L*, is a linear combination of weighted Categorical Cross-Entropy loss ($L_{wCCE}$), which attempts to enforce correct network responses (one-hot output, i.e., single-class sample membership is assumed), and regularization terms, applied to parameters of both classical neurons and L-neurons:

(12) $$\begin{array}{c} L\;=\;L_{wCCE}\;+\;\lambda_{1}L_{\alpha }\left(\alpha \right)\;+\;\lambda_{2}L_{n}\left(n\right)\;+\;\lambda_{3}L_{L2}\left(\theta ,w\right) \end{array}$$

where $L_{\alpha }\left(\alpha \right)$ and $L_{n}\left(n\right)$ are L-neuron-specific regularization terms, $L_{L2}$ is the L2 regularization [34] of parameters of conventional neurons and $\lambda_{1},\lambda_{2},\lambda_{3}$ are mixing hyperparameters.

The weighted Categorical Cross-Entropy (wCCE), which we adopt, differs from the standard formulation of CCE-loss only by the insertion of additional weights, $\omega_{c}$, that reflect within-batch class proportions, thus balancing uneven amounts of class representatives in a training set. A component of wCCE-loss that corresponds to some input sample, $x_{i}$, of a class, *c*, assumes the form: $L_{wCCE}\left(x_{i}\right)=-\omega_{c}\log y_{c}$, where $y_{c}$ is an actual network’s output at a position *c*.

**Figure 6:**
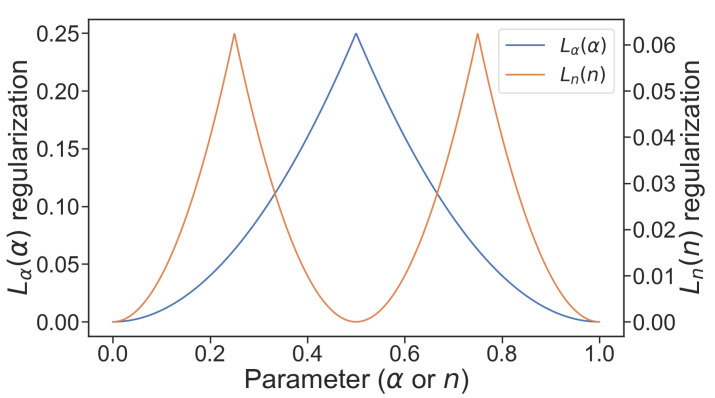
L-neuron-specific loss components.

The two L-neuron-specific loss components are defined separately for coefficients that combine *T*-norm and *T*-conorm evaluation results ($\alpha =[\alpha_{1}\cdots \alpha_{N_{L}}]$, where $N_{L}$ is a number of L-neurons) and for parameters of VCM modules (arranged in a matrix $n=[n_{1}\ldots n_{N_{L}}]$). In the former case, our objective is to maximize the interpretability of knowledge representation, i.e., we choose to favor ‘crisp’ L-neuron output expressions (either *T*-norm or *T*-conorm rather than a linear combination of the two). This can be accomplished using the following loss term:

(13) $$L_{\alpha }\left(\alpha \right)=\frac{1}{N_{L}}\sum_{i=1}^{N_{L}}{\left(\left\lfloor \alpha_{i}+\frac{1}{2}\right\rfloor -\alpha_{i}\right)}^{2}$$

where $\left\lfloor \ldots \right\rfloor$ denotes the floor operation. A plot of (13) can be seen in Figure 6.

Similarly, in the case of the second L-neuron-specific loss component, $L_{n}\left(n\right)$, which is concerned with VCM learning, we attempt to favor logical expression clarity and try to enforce explainable argument conversions (producing either unaltered, negated or neutral values). The proposed loss term is the following:

(14) $$\begin{array}{c} L_{n}\left(n_{1}\ldots n_{N_{L}}\right)=\frac{\gamma }{mN_{L}}\sum_{i=1}^{N_{L}}\sum_{j=1}^{m}U_{j}^{i}\\ U_{j}^{i}\;=\;\min\left\{{\left(n_{j}^{i}\;-\;n_{\max}\right)}^{2},{\left(n_{j}^{i}\;-\;n_{mean}\right)}^{2},{\left(n_{j}^{i}\;-\;n_{\min}\right)}^{2}\right\} \end{array}$$

where $n_{j}^{i}$ refers to current value of *j*-th input VCM control parameter of *i*-th L-neuron, γ>0 controls regularization strength and *m* is a number of L-neuron inputs. The shape of the loss component (14) is shown in Figure 6.

### 3.4. Experimental Evaluation

The objective of the experiments was to examine the classification performance of the proposed hybrid network in realistic scenarios, where knowledge of the problem is limited and only a handful of examples are available. Our primary goal was to demonstrate the proposed hybrid structure’s ability to learn effectively from examples even for highly limited datasets while benefiting from prior knowledge seamlessly introduced into the network. We also analyzed the influence of knowledge correctness on the network’s final performance, including its ability to repair that knowledge.

For benchmarking the proposed hybrid structure and the L-neurons, we chose the problem of detecting palindromes in bit strings, which is defined by a clear set of rules and enables easy manipulations of data volume and knowledge impairment. An inherent property of the palindrome detection problem is that the two underlying classes are strongly imbalanced (e.g., for 11 bit strings, there are 32 times more non-palindromes than palindromes). Therefore, the scarcity of palindrome class representatives in limited sets of examples is likely to result in overfitting if conventional machine learning approaches are used. It is well known that providing more training data results in the better performance of machine learning [35,36]. However, in practice, expanding the dataset is often costly, if not impossible. Enforcing in our experiments gradual training set size reduction complies with the perspective laid out by data efficiency evaluation, proposed by [25], and proves the utility of the proposed hybrid approach.

#### 3.4.1. Mapping Knowledge Structure onto L-Neurons

In order to build an appropriate knowledge base, we used the general rule for palindrome detection, defined as:

(15) $$\underset{n=1\cdots \lfloor \frac{1}{2}P\rfloor }{\forall }a_{n}=a_{P-n+1}$$

where bits of a palindrome *a* of length *P* (P≥3) are represented with $a_{n}$. The complementary rule can be obtained by taking the negation of the above formula. These iterative rules can be equivalently expressed by a set of first-order-logic formulas corresponding to (3) (the 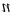 symbol is used to denote the symmetric bit in the string, that is 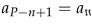): (16)
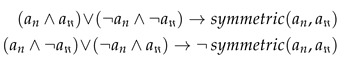

(17)
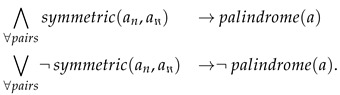


The primary advantage of the above schema is that it can be trivially mapped onto a network of proposed L-neurons (observe that for the considered case, inputs can be considered as literals, so there is no need to introduce the ‘concept extraction’ module). For a palindrome of length *P*, the first layer consists of a total of 2(P−Pmod2) logical AND operations that act on every pair of symmetrically located elements. There exists $\frac{1}{2}\left(P-P\mathrm{mod}2\right)$ such pairs.

The subsequent second layer, containing (P−Pmod2) expressions, is used to combine each pair of expressions that share the same negation pattern, that is, an even or odd number of negations, in order to determine whether it forms a symmetrical pattern. It is worth noting that Equation (16) denotes the operations performed by both layers, since the expression in the brackets denote an intermediate concept, and as such, require a separate neuron to implement them.

**Figure 7:**
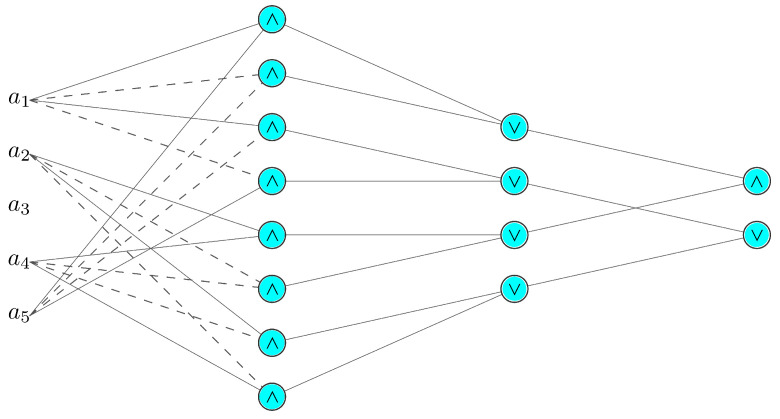
Five bit palindrome rule mapping onto a layered structure of L-neurons. The second layer of the network contains all the top-level expressions from (16), while the final layer implements (17) (passthrough connections are marked with solid lines, negations are marked with a dashed line and neutral connections are skipped for clarity).

The final, third layer consists of two rules expressed by (17), which classify a given string of bits *a* as palindrome or not palindrome. For an exemplary 5 bit palindrome problem, the mapping of the knowledge base onto the L-neuron structure is shown in Figure 7. One should note that it is not necessary to map the rules onto nodes in a layer in any particular order. As long as the correct rule structure between layers is maintained, the order of the mapping does not matter.

#### 3.4.2. Metrics

In order to measure how knowledge impairment affects classification performance, a simple method for tampering with the embedded rules was developed. Values drawn from a standard normal distribution were added at some assumed number of randomly selected entries of both α vector (L-neuron’s norm-mixing parameters) and n matrix (VCM module parameters).

To take into account the strong class imbalance, the adjusted balanced accuracy score [37] was adopted for network performance evaluation. This ensures that a perfect prediction would result in an accuracy of 1, while a random prediction would yield 0. For the considered binary case, this is equal to Youden’s J statistics [38], which is defined as:

(18) $$J=\mathrm{TPR}+\mathrm{TNR}-1=\frac{\mathrm{TP}}{\mathrm{TP}+\mathrm{FN}}+\frac{\mathrm{TN}}{\mathrm{TN}+\mathrm{FP}}-1$$

#### 3.4.3. Training Configurations

Palindrome detection in eleven bit long strings was considered throughout the experiments. All considered networks were trained on subsets comprising 1372, 1024, 512, 256, or 128 samples, i.e., providing, respectively, 67%, 50%, 25%, 12.5%, and 6.25% of the whole pool of examples. Furthermore, five levels of knowledge impairment were used: 1%, 5%, 10%, 25%, and 50%.

All of the considered structures were trained using 32-element batches for 300 epochs (in each case, the loss flattened out at least 50 epochs earlier). Additionally, every 100 epochs, the VCM’s parameters were rounded to the nearest exact value ($n_{\min}$, $n_{mean}$ or $n_{\max}$ ) corresponding to one of its modes of operation.

The hyperparameters λ, used in loss estimation (12), were optimized using the Bayesian approach, based on the adaptive Parzen–Rosenblatt estimator.

#### 3.4.4. Model Structure

The hybrid network used for palindrome detection was composed of four layers, comprising $N_{1}\;=\;30$ neurons in the first layer (made up of $N_{1L}\;=\;24$ L-neurons and $N_{1C}\;=\;6$ classical neurons), $N_{2}\;=\;15$ neurons in the second layer ($N_{2L}\;=\;12$, $N_{2C}\;=\;3$), $N_{3}\;=\;4$ neurons in the third one ($N_{3L}\;=\;2$, $N_{3C}\;=\;2$), and 2 classical neurons in the output layer. The amounts of L-neurons in subsequent layers were chosen to provide 20% excess over the minimum required to correctly embed the domain knowledge for the considered case. The adopted overhead was determined empirically to enable a different, albeit still valid, set of rules was arrived at after training settled down.

To ensure the supremacy of knowledge in decision making, initial couplings between the knowledge sub-network and output-layer neurons were nine times stronger than couplings with the conventional neural sub-network.

To assess the quality of the proposed hybrid network, we compared its performance with classification results produced by three reference architectures. The first one was a network composed exclusively of L-neurons, with the structure identical to the knowledge sub-network of the proposed hybrid architecture ($N_{1}\;=\;N_{1L}\;=\;24$, $N_{2}\;=\;N_{2L}\;=\;12$ and $N_{3}\;=\;N_{3L}\;=\;2$). The second and the third reference structures were purely neural architectures of two different complexities: matching the layer widths of the whole hybrid network ($N_{1}\;=\;N_{1C}\;=\;30$, $N_{2}\;=\;N_{2C}\;=\;15$ and $N_{3}\;=\;N_{3C}\;=\;2$) and matching only its neural sub-network ($N_{1}\;=\;N_{1C}\;=\;6$, $N_{2}\;=\;N_{2C}\;=\;3$ and $N_{3}\;=\;N_{3C}\;=\;2$). To maximize performance of the reference, purely neural structures, ReLU activations were set for all of their units.

## 4. Results and Discussion

**Figure 8:**
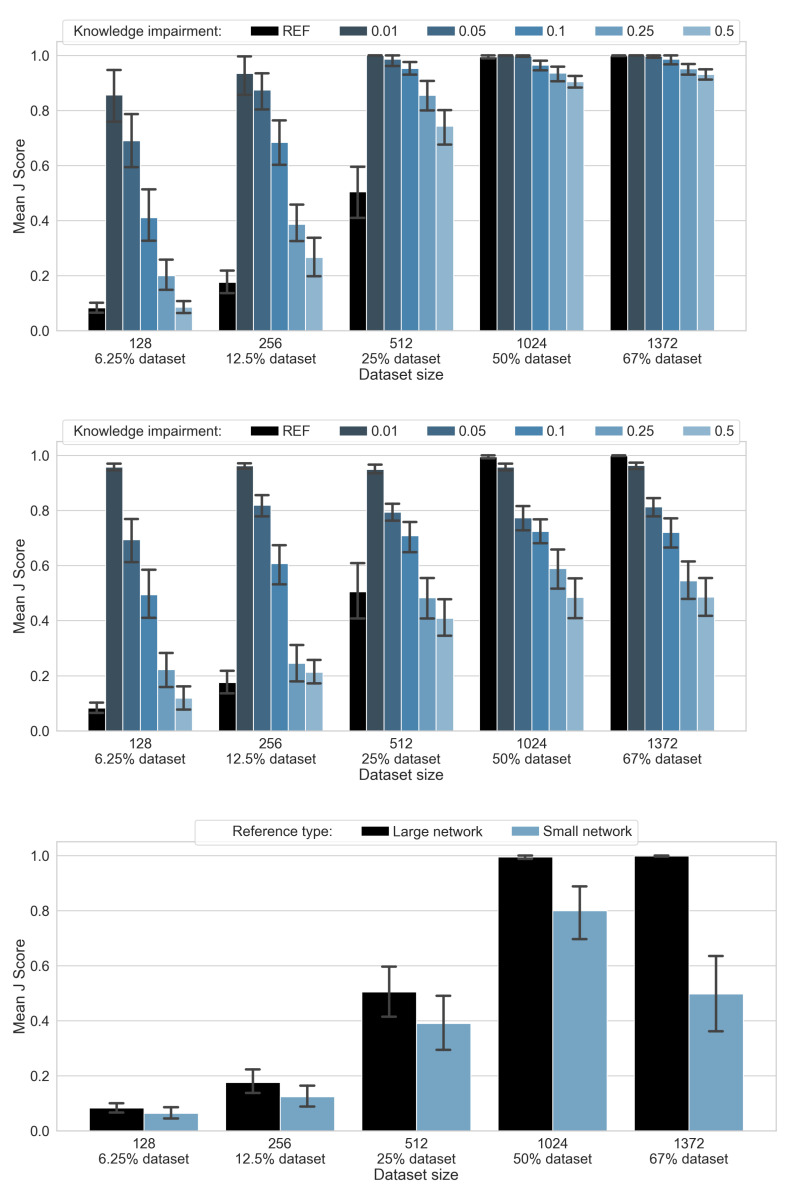
Adjusted classification accuracy scores for the hybrid network, L-neuron-only network and the two reference classical networks for different knowledge impairment levels and different training set volumes. (**Top**) Hybrid network performance ($J^{H}$). (**Middle**) L-neuron-only network performance ($J^{L}$). (**Bottom**) Performance of the reference neural networks. "REF" indicates performance of the reference, "larger" neural network. Confidence intervals of 95% are marked with whiskers.

The results of palindrome detection experiments prove that both hybrid and L-neuron-only structures are capable of outperforming classical neural networks in the case of limited training dataset sizes, even when available knowledge is impaired (as shown in Figure 8, top, and Figure 8, middle, respectively). These results are also summarized in Table 1.

For the hybrid structure, this observation holds for training set volumes reduced by up to 25% of the complete dataset (up to 512 out of 2048 examples) and for knowledge impairment levels up to 50%, whereas for the purely L-neuron-based structure, this remains true for sizes up to 12.5% of the original volume. It is worth noting that the reference, purely a neural network of the same size (thus, of similar capacity) as the hybrid structure performs noticeably better than the second, smaller reference neural network (see Figure 8, bottom). Therefore, results obtained for the ’larger’ neural structure were considered for comparisons presented in Figure 8, top, and Figure 8, middle.

### 4.1. L-Neuron Network Evaluation

**Figure 9:**
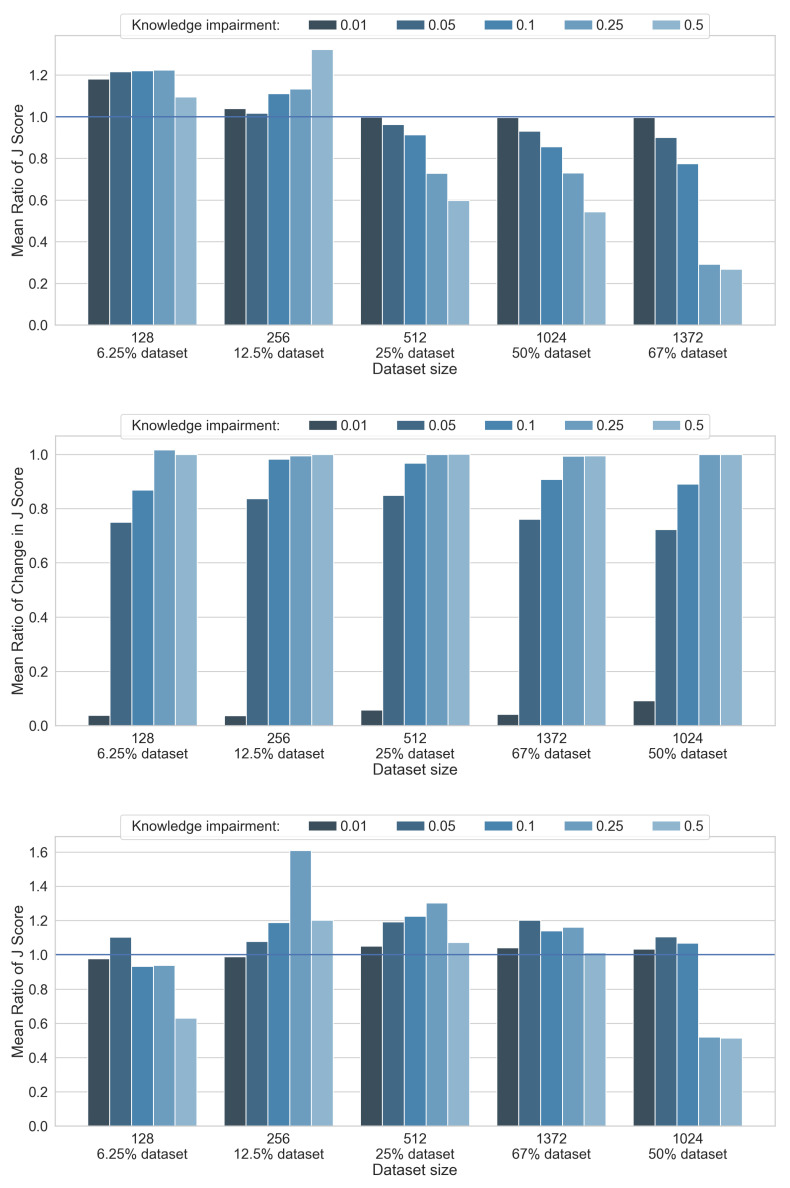
Relative classification accuracy scores for the knowledge subnetwork of hybrid network, learned performance of L-neuron-only network and the comparison between the performance of knowledge subnetwork of hybrid network and L-neuron-only network. (**Top**) Relative performance $J^{H_{L}}/J^{H}$. (**Middle**) Relative performance: $\left(J^{L}-J^{L_{0}}\right)/J^{L}$. (**Bottom**) Relative performance: $J^{H_{L}}/J^{L}$. $J^{H_{L}}$ denotes performance of hybrid network’s knowledge sub-network, $J^{L_{0}}$—initial performance of L-neuron-only network. Confidence intervals of 95% are marked with whiskers.

The logical L-neuron-only structure itself exhibits a noticeable level of trainability, as presented in Figure 9, middle. As it could be anticipated, when prior knowledge of good quality is injected into the network (only 1% impairment), only slight training-induced improvements are sufficient to achieve a high final performance. For models with more than 5% knowledge impairment, the majority of final performance can be attributed to training (spectacular jumps in classification accuracy after training can be seen in Figure 9, middle). This proves that a network composed only of L-neurons is capable of improving its initial, even severely impaired knowledge. However, if the amount of training data increases (which, for the considered problem, occurs in around 512 samples), L-neuron-based architecture is not able to match the performance of the purely neural reference when being fed with severely impaired knowledge.

Regarding the issue of violating logic principles that might occur as an effect of L-neuron training, due to the regularization provided through the adopted loss function, rounding to the pure logic outcome was applied rarely, only every 100 epochs. In all cases, it only resulted in minor parameter alterations, i.e., deviations from what logic expects, were minor.

### 4.2. Knowledge Repair

**Figure 10:**
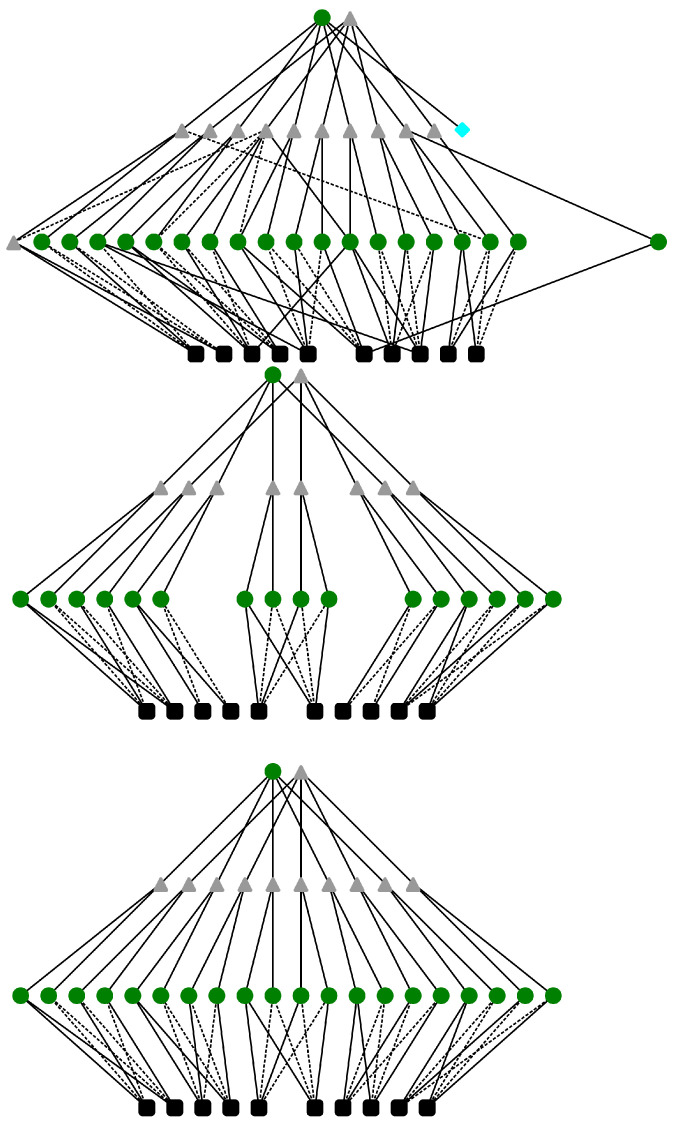
Comparison of L-neuron structures in a purely knowledge-based model for 0.05 impairment and the 1024 element dataset. Dashed lines indicate complemented connection, while continuous lines depict passthrough connections (neutralizing connections are skipped for the sake of clarity). Circular green nodes indicate dominance of logical AND (α→1), whereas triangular grey nodes indicate dominance of OR (α→0). Black squares designate inputs, while the blue rhombus indicates an L-neuron with no inputs. Neurons in each layer are drawn in the same order in all graphs. Neurons that do not influence the output value are removed to further improve readability. The inputs are placed in a paired order (1, 11, 2, 10, 3, 4 (not shown), 9, 5, 8, 6, 7) in order to increase readability. (**Top**) Initial, impaired rule layout. (**Middle**) Rule layout obtained after training. (**Bottom**) A desired “perfect” rule structure.

Although the final structure produced by training (Figure 10) does not fully recover the correct logical palindrome-detecting expression, it is cleaned from malformed or redundant terms. In the presented example, one can observe a significant reduction in the number of connections in the graph. Additionally, the “empty” L-neuron with no inputs is eliminated during optimization.

Observations of the resulting structures across all experiments indicate a tendency of optimizers towards disabling misconfigured L-neurons from the structure (node disabling is achieved by neutralizing their inputs by corresponding VCM modules).

It is notable that in the presented case, the last neuron in the first layer was neutralized, and its role was filled by correctly rewiring a different neuron to implement the missing rule. This observation provides a further explanation of the limited learning capabilities of the L-neuron-only structure. The optimization process appears to favor the removal of infringing rules over correcting them. As a result, the final structure is fully compliant with the information provided by the samples but is incomplete, and as such, it cannot reach perfect performance.

### 4.3. Hybrid Network Evaluation

The proposed hybrid architecture, where the L-neuron-only network is supplemented with the classical neural structure, enables a leap in performance (Figure 8, top), providing almost perfect classification performance for a wide range of training set sizes and initial knowledge impairment levels. It is noteworthy to observe that for a limited size of a training set (up to 12.5%), the final performance of the hybrid structure, comparable to the one offered by the L-neuron-only network, is ensured mainly by its knowledge-based part (see Figure 9, top). This smoothly changes in favor of the conventional neural sub-network, as the amount of available training data increases. Additionally, the decision-making role of the neural sub-network of the hybrid structure further increases as initial knowledge impairment levels become higher.

To assess how the presence of the neural sub-network affects the outcome of knowledge sub-network training, we confronted classification accuracy provided by the knowledge module, extracted after training completion from the hybrid network, with scores produced by separately trained L-neuron-only network. As can be seen in Figure 9, bottom, the knowledge sub-network of the proposed hybrid structure mostly trains better than its L-neuron-only counterpart. Only for high knowledge impairment levels (25% or more) and extreme—either very small or very large—amounts of data, L-neuron-only structure training is more effective. These results indicate that the proposed hybrid model maintains the ability to correctly train its knowledge sub-network, enabling interpretability of the resulting, corrected knowledge.

## 5. Conclusions

The concept proposed in the paper was aimed to enable intelligent data analysis using smart sensors, where compensating learning by knowledge reduced a need for having large training datasets and highly complex neural networks. We have shown that the presented idea of combining knowledge and learning may result in better problem-solving performance. Mutual benefits of merging knowledge and learning have been demonstrated through knowledge impairment correction and data-scarcity compensation. In the former case, a logical reasoning structure with an impaired inference path has been appropriately amended through a subnetwork of regular neurons. In the latter case, the proposed way of injecting prior knowledge has enabled complexity reduction in a neural structure, as regular neurons only need to learn the missing pieces of problem–solution principles.

The performance of the *hybrid* model proves our intuitive expectation that the proposed hybrid structure would be capable of leveraging information contained in the provided dataset in order to compensate for erroneous rules. Such behavior could be interpreted as an analog to the two systems described by Kahneman [39]. The L-neurons may be thought of as the more logical and rigid System 2, whilst the classical nodes could be considered to be akin to the intuitive System 1.

The primary contribution of this work is the concept of the L-neuron, not only capable of representing knowledge in the form of logical rules but also of being trained using classical backpropagation methods.

Another essential contribution is that our approach does not need a specific part of knowledge to be fully and correctly defined. We have shown that the knowledge sub-network of the proposed hybrid structure, as well as the L-neuron-only structure, are capable of partial self-correction over the course of training.

The third contribution is that the embedded knowledge maintains a high degree of comprehensibility even after training and is not overpowered by the added classical neurons. This opens up a route towards explainable neural models, which, due to their extreme number of parameters, have thus far been treated primarily as black-box models.

Finally, since the underlying operators used for embedding knowledge have been adopted from fuzzy logic, the system is also capable of carrying out fuzzy inference. The augmentation of the proposed models with a more general method for input fuzzification would allow our proposed approach to operate in any domain. Such augmentation was deemed to be out of the scope of this article. Nevertheless, it is a promising area of future work.

## Figures and Tables

**Table 1:** Aggregated values of the *J* statistics for the L-neuron-only and hybrid architectures evaluated on test subsets. The size of considered datasets is shown in row (1), rows designated with (2) are the mean values of *J* statistics for 25 trials and those designated with (3) present performance gains with respect to the ‘larger’ reference neural network. Labels *ki* indicate levels of knowledge impairment. Gains in performance are marked in bold font.

	ki = 0.01					ki = 0.05					ki = 0.1					ki = 0.25					ki = 0.5				
(1)	**128**	**256**	**512**	**1024**	**1372**	**128**	**256**	**512**	**1024**	**1372**	**128**	**256**	**512**	**1024**	**1372**	**128**	**256**	**512**	**1024**	**1372**	**128**	**256**	**512**	**1024**	**1372**
	**L-neuron-only**																								
(2)	0.96	0.96	0.95	0.96	0.96	0.69	0.82	0.79	0.77	0.81	0.49	0.61	0.71	0.72	0.72	0.22	0.25	0.48	0.59	0.54	0.11	0.20	0.41	0.48	0.49
(3)	**0.87**	**0.79**	**0.45**	−0.04	−0.04	**0.61**	**0.64**	**0.29**	−0.22	−0.19	**0.41**	**0.43**	**0.20**	−0.27	−0.28	**0.14**	**0.07**	−0.02	−0.41	−0.45	**0.03**	**0.02**	−0.10	−0.51	−0.51
	**Hybrid**																								
(2)	0.83	0.94	1	1	1	0.69	0.87	0.99	1	1	0.41	0.68	0.95	0.97	0.99	0.19	0.39	0.86	0.94	0.95	0.08	0.24	0.74	0.91	0.93
(3)	**0.74**	**0.76**	**0.50**	**0.01**	0	**0.61**	**0.70**	**0.48**	0	0	**0.33**	**0.51**	**0.45**	−0.03	−0.01	**0.11**	**0.21**	**0.35**	−0.06	−0.05	−0.01	**0.06**	**0.24**	−0.09	−0.07

## Data Availability

Not applicable.
